# Exploring suicide risk among female inpatients with eating disorders: a clinical perspective

**DOI:** 10.1007/s40519-025-01768-7

**Published:** 2025-07-21

**Authors:** Massimo Pasquini, Salvatore Sarubbi, Elena Rogante, Annalisa Maraone, Irene Pinucci, Flavia Boccardi, Paola di Girolamo, Isabella Berardelli, Marco Innamorati, Maurizio Pompili

**Affiliations:** 1 Department of Human Neurosciences, Sapienza University of Rome, Rome, Italy; 2 Department of Neurosciences, Mental Health, and Sensory Organs, Faculty of Medicine and Psychology, Suicide Prevention Centre, Sant’Andrea Hospital, Sapienza University of Rome, Rome, Italy; 3 Department of Human Sciences, European University of Rome, Rome, Italy

**Keywords:** Eating disorders, Mental pain, Suicidal ideation, Suicidal behaviors, Demoralization

## Abstract

**Purpose:**

Patients with eating disorders show an elevated suicide risk compared to the general population. Adverse childhood experiences, depressive symptoms, and mental pain, often accompanied by hopelessness and demoralization, may increase this risk. This study aims to explore suicidal ideation and self-injurious behaviors in a heterogeneous sample of ED patients, and their association with childhood trauma and negative mental states.

**Methods:**

101 female patients were recruited from the inpatient and day hospital ED units at Policlinico Umberto I, Rome. Assessments included comorbid psychiatric diagnosis, suicide risk, mental pain, childhood trauma, depressive symptoms, hopelessness, and demoralization.

**Results:**

Suicidal ideation in the past month was significantly associated with trait and state-level mental pain, self-reported sexual abuse, depression, hopelessness, and loss of meaning. In the multivariate model, only childhood sexual abuse and loss of meaning remained significantly associated. Self-injurious behaviors in the past 3 months were associated with trait-level psychache, self-reported sexual and physical abuse, and depression severity, however significantly associated with self-injurious behaviors in the multivariate model.

**Conclusions:**

Findings highlight the importance of assessing childhood trauma, especially sexual abuse, a low sense of meaning in life, depressive symptoms, and psychache in patients with EDs to improve suicide prevention strategies with specific interventions.

*Level of evidence* III—Evidence obtained from well-designed cohort or case–control analytic studies.

## Introduction

Eating disorders (EDs) are major psychiatric conditions characterized by abnormal eating behaviors and preoccupation with food, frequently associated with body weight and shape concerns [1]. The complexity of these disorders stems from the interplay of genetic, psychological, and sociocultural factors, underscoring the need for a comprehensive understanding of effective treatment. All EDs, and especially anorexia nervosa (AN), are associated with medical complications and increased mortality, with suicide being one of the significant causes of death [2, 3]. Indeed, AN patients are 31 times more likely to die by suicide, and diagnoses of bulimia nervosa (BN) and binge eating disorder (BED) are associated with both increased suicidal ideation and suicide attempts compared to controls [4, 5]. The considerable comorbidity between EDs and suicide behaviors, including non-suicidal self-injury (NSSI) and suicidal ideation, underlines the importance of investigating key risk factors [6–8]. Suicidal ideation and suicidal behaviors often represent a maladaptive coping strategy in response to emotional dysregulation and psychological pain, which are usually relevant in individuals with EDs [9]. Several factors may influence suicidal ideation and behaviors in individuals with EDs, for example, distal factors such as adverse childhood experiences are highly comorbid with EDs and have strong associations with a higher suicide risk [10, 11]. Notably, child sexual abuse is known to increase the risk for both suicidal ideation and suicide attempts, and NSSI during adulthood [12–14]. Proximal emotional and cognitive mental states are other well-known risk factors for suicidal ideation, suicide behaviors, and NSSI. Depressive symptoms have often been associated with suicidal ideation and suicide attempts in individuals with EDs [15, 16]. Mental pain, as the mental state of individuals experiencing a state of inner turmoil and perturbation [17], has been suggested as crucial in contributing to cognitive tunneling and leading to a suicidal act seen as the only option available to stop the unbearable pain the individual is experiencing. Some studies [18, 19] have observed that mental pain is higher in patients with EDs than in controls and hypothesized that it might have a role in determining suicide risk in this population. Hopelessness, characterized by negative expectations toward oneself and one’s future [20], represents a crucial risk factor for suicide in several theoretical models and may be exacerbated by the lack of self-esteem, control, and identity associated with eating disorders [21]. The presence of mental pain and hopelessness could lead to the development of a demoralization syndrome [22], described as a syndrome separated from depression and characterized by helplessness, a sense of failure, loss of hope, and loss of meaning, that has been frequently associated with increased suicide risk [23–25]. Such feelings may interact with the emotional and psychological distress experienced by ED patients and influence suicide risk. Indeed, according to several studies [26, 27], the meaning in life is lower in those with EDs than in non-clinical populations, and this construct is a significant predictor of the eating disorder psychopathology, NSSI, and suicidal ideation.

According to the above literature review, the primary purpose of the present study was to investigate the presence and severity of suicidal ideation and self-injurious behaviors, a category of behaviors including both suicidal and non-suicidal behaviors in a heterogeneous sample of patients with eating disorders and associated factors. We analyzed whether distal factors, such as childhood trauma and negative mental states (i.e., self-reported depression, hopelessness, mental pain, and demoralization), were associated with higher recent suicidal ideation severity and the risk of acting self-injurious behaviors. We also analyzed differences between AN patients and a subsample of patients that included those with BN and BED. We hypothesized that the presence of more severe suicidal ideation (and the presence of self-injurious behaviors) was associated with comorbidity with mood disorders, cluster B personality disorders, and more severe mental pain, hopelessness, and demoralization. Furthermore, we also hypothesized an association between childhood traumas and suicidal ideation and self-injurious behaviors.

## Materials and methods

The participants were 101 female patients. The mean age of the sample was 22.26 years (SD = 6.08 years; Age range = 18/47 years). Most participants were single (96%) and employed or students (90%)—Table 1 lists demographic and clinical characteristics of the sample. Participants were enrolled from the inpatient ward and the day hospital for eating disorders at Policlinico Umberto I of Rome and had been hospitalized between September 1, 2019 and February 29, 2024.

**Table 1:** Sociodemographic and clinical characteristics of the sample

	Frequency	Percent
Age – M|SD	22.26	6.08
Marital status		
Single	97	96.0
Married	4	4.0
School		
≤ 8 years	23	23.0
≤ 13 years	59	59.0
≥ 16 years	18	18.0
Job		
Employed or students	90	90.0
Unemployed	10	10.0
DSM-5 major diagnosis		
AN	39	42.9
BN	29	31.9
BED	9	9.9
Eating disorder NOS	14	15.4
Comorbid diagnosis		
None	64	65.3
Mood disorder	29	29.6
Psychotic disorder	1	1.0
Anxiety disorder	4	4.1
Comorbid cluster B personality disorder	8	8.3
Onset age – M|SD	15.71	3.09
Psychiatric diagnosis in family members	33	30.4
Lifetime suicidal ideation severity – M|SD	1.22	1.52
Lifetime self-injurious behavior	29	30.5
Lifetime suicide attempt	3	3.2
Lifetime non-suicidal self-injurious behaviors	28	29.8
Last month suicidal ideation severity – M|SD	0.43	1.07
Last 3 months self-injurious behavior	11	11.6
Last 3 months suicide attempt	0	0.0
Last 3 months non-suicidal self-injurious behaviors	11	11.7

*AN* Anorexia Nervosa, *BN* Bulimia nervosa, *BED* Binge Eating Disorder, *NOS* Not Otherwise Specified

Upon closer inspection of the P.I. (first author), patients were assessed for psychiatric diagnoses according to the Diagnostica and Statistical Manual of Mental Disorders, Fifth Edition (DSM-5) criteria [28], and these diagnoses were confirmed by the Structured Clinical Interview for DSM-IV Axis I disorders (SCID-I) [29], via psychiatric interviews and the administration of psychometric instruments. The inclusion criterion was to be between 18 and 65 years of age (subjects over 65 were included only if physically fit, that is, they lacked medical comorbidities that reduced quality of life, caused impairments, etc.). Exclusion criteria were being unwilling to participate or denial of informed consent and having neurologic diseases (e.g., dementia, Parkinson’s disease, epilepsy, etc.), cognitive impairments, and language difficulties. All participants received a comprehensive explanation of the study procedures and signed a written informed consent form. All participants were free to withdraw from participating in the study at any time. The questionnaires were administered by two medical doctors undergoing specialist training in psychiatry under the supervision of senior consultant psychiatrists. The participants were treated in accordance with the ethical principles outlined in the Declaration of Helsinki. The local ethical review board approved this study.

### Measures

All the patients were administered a socio-anamnestic form to collect data on sex, age, marital status, job condition, diagnosis, age at illness onset, and comorbidities. Patients were assessed for psychiatric diagnoses according to the Diagnostic and Statistical Manual of Mental Disorders, 5th edition [28]. Furthermore, suicidal ideation severity, suicide attempt, and NSSI were evaluated using the Columbia-Suicide Severity Rating Scale [30]. The C-SSRS is a semi-structured interview to assess suicidal ideation, behavior, and NSSI in individuals aged 12 years or older. The interview focuses on both the lifetime period and the recent period (last month for suicidal ideation and the previous 3 months for suicidal behaviors).

The Psychache Scale (PAS) [31] is a self-report scale comprising 13 items that evaluates the presence and the frequency of psychological pain on a 5-point Likert scale. The PAS has demonstrated satisfactory reliability and validity [31, 32]. The Italian version was used in previous studies in both clinical and non-clinical samples [33–35]. In the present sample, Cronbach’s alpha was 0.95.

The Visual Analog Scale of psychical and psychological pain (VAS) [36] is a brief questionnaire that assesses the worst, usual, and current levels of mental pain on a dimensional scale from 0 (none) to 10 (maximum possible pain). In the present sample, Cronbach’s alpha was 0.83.

The Childhood Trauma Questionnaire (CTQ) [37] is a 28-item self-report questionnaire assessing emotional (EA), physical (PA), and sexual abuse (SA) and emotional (EN), and physical neglect (PN). For each item that begins with the anchor “When I was growing up”, the respondents indicate the frequency of events using a 5-point Likert scale ranging from “1 = never true” to “5 = very often true”. The questionnaire demonstrated good psychometric properties [38, 39]. In the present sample, Cronbach’s alphas were 0.81 for EA, 0.87 for PA, 0.88 for SA, 0.86 for EN, and 0.31 for PN.

Beck Depression Inventory (BDI) [40] is a 21-item self-report instrument that evaluates the severity of depressive symptoms during the previous 14 days. Each item is scored from 0 to 3, assessing symptom severity, with a total score ranging from 0 to 63. Internal consistency and concurrent validity have been documented in both clinical and non-clinical samples [41]. In the present sample, Cronbach’s alpha was 0.90.

The Beck Hopelessness Scale (BHS) [42, 43] is a 20-item self-report measure of hopelessness. Higher scores indicate more severe hopelessness. Several international studies reported good psychometric properties of the BHS and suggested satisfactory ability in predicting subsequent suicide behavior, and general health and social functioning [44, 45]. A score of 9 or higher could detect patients at risk for suicide [46, 47]. Validation studies have been conducted in Italy on samples of medical patients, university students, and psychiatric inpatients, demonstrating satisfactory psychometric properties [45]. In the present sample, Cronbach’s alpha was 0.91.

Demoralization index [48] is a 24-item self-report scale to assess demoralization level based on five dimensions (Loss of meaning, Distress/Dysphoria, Disheartenment, Helplessness, Sense of Failure). The items are rated on a Likert scale ranging from 0 (not at all) to 4 (very much), with higher scores indicating greater demoralization. Previous studies demonstrated robust psychometric properties [49]. In the present sample, Cronbach’s alpha was 0.91 for Loss of meaning (LM), 0.78 for Distress/Dysphoria (Distress), 0.89 for Disheartenment (DIS), 0.79 for Helplessness (HELP), and 0.83 for Sense of Failure (FAIL).

### Statistical analysis

All analyses were performed using the Statistical Package for the Social Sciences (SPSS) version 19.0 (IBM SPSS 19.0, Armonk, NY). We included in the self-injurious behavior category any non-suicidal self-injurious behavior and any suicidal behavior (i.e., suicide attempts, preparatory acts, and aborted or interrupted suicide behaviors).

One-way Fisher’s exact tests (FET) and t tests were used to compare AN patients and a composite subsample that included BN and BED patients.

A series of single-variable generalized linear models (GLM) with robust estimators was used to assess the association between the predictors and the criterion (i.e., last month suicidal ideation severity and the presence of self-injurious behaviors in the previous 3 months). Only the CTQ and the demoralization index dimensions were included as a block in two GLM models.

Considering the low reliability of CTQ PN, this dimension was excluded from the analyses. All the predictors significantly associated with suicidal ideation severity in the last month (or the presence of self-injurious behavior in the last 3 months), after correction for multiple testing, were included in a final GLM model. Odds ratios were reported as measures of association. Tests are considered significant for p < 0.05. A post hoc power analysis was conducted to determine whether the sample size was sufficient for testing our hypothesis. The analysis indicated satisfactory power (1 – β = 0.82) to detect a medium effect size (f^2^ = 0.15), suggesting that the model was sufficiently sensitive to detect statistically significant effects.

## Results

### Characteristics of the sample

More than 74% of the patients had either AN or BN as a primary eating disorder (see Table 1), and 9.9% had BED. Around 30% of the patients had comorbid mood disorders, and 8.3% had a comorbid cluster B personality disorder.

No patients had attempted suicide in the last 3 months, while 11 reported at least one type of self-injurious behavior (Table 1).

Table 2 lists scores on psychological tests. More than 80% of the patients reported moderate-to-severe self-reported depression, and 58% reported severe hopelessness. Around 7% of the sample reported passive suicide ideation and death wishes, and more than 11% reported active suicide ideation, with 4% of them reporting some intent to act.

**Table 2:** Psychological testing results

Variables	Mean	Std. Deviation
PAS	43.27	11.96
VAS	19.99	6.57
EA	10.01	4.62
PA	6.23	3.17
SA	5.74	2.64
EN	12.00	4.78
BDI	28.44	11.78
BDI ≥ 18	83	82.2
BDI (without item #9 assessing suicidal ideation)	27.90	11.38
BHS	10.02	5.56
BHS ≥ 9	58	58.0
LM	7.17	5.15
Distress	13.19	4.06
DIS	15.50	5.47
HELP	8.84	3.68
FAIL	8.91	3.65

*PAS* Psychache Scale, *VAS* Visual Analog Scale, *EA* Emotional Abuse, *PA* Physical Abuse, *SA* Sexual abuse, *BDI* Beck Depression Scale, *BHS* Beck Hopelessness Scale, *LM* Loss of Meaning, *DIS* Disheartenment, *HELP* Helplessness, *FAIL* Sense of Failure

When comparing AN patients and BN/BED patients, groups of patients differed only for the presence of Cluster B personality disorder (0% vs. 13.9%; FET p = 0.023) although the difference was not significant anymore after correction for multiple testing. Differences between groups of patients on scores for psychological testing were not significant, even before correction for multiple testing (p > 0.05), and remained non-significant after correction for multiple testing (p > 0.05).

Finally, groups did not differ for suicidal ideation severity (Lifetime: 1.08 + 1.362 vs. 1.19 + 1.67, t71 = -0.32, p = 0.75; Last month: 0.47 + 1.01 vs. 0.42 + 1.16, t72 = 0.23, p = 0.82), and self-injurious behaviors (Lifetime: 21% vs. 26%, FET p = 0.42; Last 3 months: 11% vs. 11%, FET p = 0.60).

### Factors associated with suicidal ideation severity in the past month

Single-variable GLMs indicated six variables significantly associated with the severity of suicidal ideation in the last month, even after correction for multiple testing (see Table 3). More severe suicidal ideation was associated with a more severe trait- and state-level mental pain, self-reported sexual abuse, depression severity, hopelessness, and higher loss of meaning. All variables significant at the single-predictor GLM models were entered into a multivariate model with suicidal ideation in the last month as the criterion (likelihood ratio χ^2^ = 34.30, p < 0.001) (see Table 3). Only CTQ SA (Wald χ^2^ = 7.20, p = 0.007) and LM (Wald χ2 = 4.81, p = 0.03) were significantly associated with the severity of suicidal ideation in the last month. Patients with more severe childhood history of sexual abuse (compared to other patients) were 1.10 times (odds ratio range = 1.03/1.18) more likely to have more severe suicidal ideation in the last month. Patients with higher LM scores were 1.09 times (odds ratio range = 1.01/1.17) more likely to have more severe suicidal ideation in the last month.

**Table 3:** Generalized Linear Models (criterion: Last month suicidal ideation severity)

Models		Wald Chi-Square	Significance	Wald Chi-Square	Significance
1	**Age**	0.64	0.42	–	–
2	**AN**	0.001	0.97	–	–
2	**BN**	0.38	0.54	–	–
3	**Mood disorders**	2.73	0.10	–	-
4	**Cluster B personality disorder**	3.25	0.07	–	–
5	**PAS**	9.75	**0.002**	1.52	0.22
6	**VAS**	7.99	**0.005**	0.45	0.50
7	**EA**	0.96	0.33	–	–
7	**PA**	0.32	0.57	–	–
7	**SA**	10.12	**0.001**	**7.20**	**0.007**
7	**EN**	2.03	0.15	–	–
8	**BDI (without item #9 assessing suicidal ideation)**	15.22	** < 0.001**	0.77	0.38
9	**BHS**	13.76	** < 0.001**	0.005	0.95
10	**LM**	9.72	**0.002**	**4.81**	**0.03**
10	**Distress**	0.01	0.93	–	–
10	**DIS**	0.14	0.71	–	–
10	**HELP**	0.43	0.52	–	–
10	**FAIL**	0.05	0.83	–	–

In bold results significant for p ≤ 0.05. Multiple testing correction: 0.05/10 = 0.005

### Factors associated with self-injurious behaviors in the past 3 months

Single-variable GLMs indicated five variables significantly associated with self-injurious behaviors in the last 3 months although only three variables were significant after correction for multiple testing (see Table 4). The presence of self-injurious behaviors in the last 3 months was associated with more severe trait-level psychache, self-reported sexual and physical abuse (the last variable was not significant after multiple testing), and depression severity. The three significant variables at the single-predictor GLM models were entered in a multivariate model with self-injurious behaviors in the last 3 months as a criterion (likelihood ratio χ^2^_3_ = 12.13, p = 0.007), and no variable was significantly associated with the presence of self-injurious behaviors in the last 3 months (p > 0.05).

**Table 4:** Generalized Linear Models (criterion: Presence of self-injurious behaviors in the last 3 months)

Models		Wald Chi-Square	Significance
1	**Age**	0.74	0.39
2	**AN**	0.01	0.94
2	**BN**	0.30	0.58
3	**Mood disorders**	0.01	0.93
4	**Cluster B personality disorder**	5.02	**0.03**
5	**PAS**	10.91	**0.001**
6	**VAS**	1.25	0.26
7	**EA**	3.38	0.07
7	**PA**	4.99	**0.03**
7	**SA**	11.24	**0.001**
7	**EN**	1.74	0.19
8	**BDI**	8.23	**0.004**
9	**BHS**	3.26	0.07
10	**LM**	0.02	0.90
10	**Distress**	0.12	0.73
10	**DIS**	0.30	0.59
10	**HELP**	0.25	0.62
10	**FAIL**	0.03	0.86

## Discussion

This study explored the association between hopelessness, mental pain, depressive symptoms, childhood trauma, and demoralization with suicide risk in a heterogeneous sample of patients with EDs. The results showed that there were no differences between groups according to the diagnosis of ED (AN vs BN/BED). This finding might be explained by considering the low number of patients included in the analysis. However, considering the whole sample, patients reporting childhood sexual abuse and higher loss of meaning were more likely to report more severe suicidal ideation in the past month. Other studies [13, 50, 51] observed an association between childhood sexual abuse and suicidal ideation, hypothesizing that it might be related to emotion dysregulation, higher depressive symptoms, and feelings of hopelessness frequently associated with EDs [52, 53]. For example, Bahk et al. [54] have observed that only childhood sexual abuse directly predicted suicidal ideation. In contrast, other kinds of childhood maltreatment were indirectly associated with suicidal ideation through the mediation of different variables.

Loss of meaning in life, a construct included in the concept of demoralization, has also been frequently associated with suicidal ideation [55, 56], considered a transnosographic feature [25] and may heighten known risk factors for suicide, such as hopelessness and loneliness. In this context, a study by Kleiman and Beaver [57] has shown that the presence of meaning in life predicts lower suicidal ideation and lower lifetime odds of a suicide attempt; the authors argued that meaning in life increases suicide resiliency above and beyond the effects of low levels of risk factors and high levels of protective factors and suggested to intervene on this facet to decrease suicide risk.

Moreover, relatively to self-injurious behavior, in our sample, 3.2% of the patients had a lifetime suicide attempt, and none attempted suicide in the previous 3 months. These data are similar to those from recent studies [58, 59], for example, in a sample of patients with AN, Longo et al. [59] reported that 9.7% had a lifetime SA, while an extensive retrospective cohort study by Cliffe et al. [58] registered a lifetime prevalence of SA around 6.7% in a sample of patients with AN, BN, and Eating disorders Not Otherwise Specified (EDNOS). On the other hand, our data are lower than those from other studies where the prevalence of suicide attempts in eating disorders was around 20% [8, 60], probably indicating a lower suicide risk in this population of ED patients. However, lifetime non-suicidal self-injurious behavior was reported in around 30% of the patients, and in the last 3 months, non-suicidal self-injurious behavior was reported in more than 11% of the sample.

Regarding predictors of self-injurious behaviors in the last 3 months, our findings have shown that, at the single-predictor GLM, three variables were significant (depression severity, trait-level psychache, and sexual abuse) although none of them remained significant in the final GLM. Scientific literature has repeatedly highlighted the association between depression, psychache, sexual abuse, and suicide risk. For example, the multicenter study by Pompili et al. [61], on a large sample that also included 231 patients with ED, has shown that patients with a recent suicide attempt reported higher mental pain and that the relationship between childhood trauma and suicide risk was mediated by mental pain. Moreover, Pompili et al. [34] highlighted a clinical phenotype of patients with high levels of depression and mental pain related to a severe pattern of suicide risk, characterized by high hopelessness, presence of suicidal ideation, and previous suicide behaviors.

Our findings underline the need for considering adverse childhood experiences, in particular sexual abuse, a low sense of meaning in life, depressive symptoms, and psychache in patients with EDs to improve suicide prevention strategies with specific interventions. It is fundamental to thoroughly assess and screen for potential risk factors to design personalized approaches that consider mental pain, early traumatic experiences, hopelessness, and demoralization as each one of these variables has been associated with suicide risk and must be included in tailored interventions. Furthermore, it would be helpful to address the transversal mechanisms that are common in patients with EDs and at risk for suicide or NSSI. From this perspective, it is crucial to identify and assess emotion regulation strategies, impulsiveness, affect lability, and loneliness, and then implement targeted interventions.

Future studies should adopt a longitudinal design to highlight individual pathways and clarify cause–effect relationships. Such studies would also allow the identification of more accurate predictors that can increase suicide risk vulnerability. Furthermore, it would be beneficial conducting interventional studies to explore the effectiveness of specific therapeutic strategies to reduce suicide risk and enhance long-term clinical outcomes for patients with EDs.

## Strengths and limits

The present study has a multidimensional approach to evaluate suicide risk in patients with eating disorders that provides an in-depth vision of the complexity of suicide, through the assessment of distal factors, such as childhood maltreatment, and proximal factors, such as depression, psychache, hopelessness and demoralization. Moreover, the measures used to assess psychometric variables are reliable and validated international instruments with high internal consistency, which confers an additional strength to the results. Finally, the associations reported between the variables are relevant from a clinical point of view for prevention and assessment of suicide risk in patients with eating disorders, with a focus on the design of personalized interventions. It is also important to note the study’s limitations: the sample is relatively small and may not be sufficiently representative, limiting the study results’ generalizability; moreover, the study's cross-sectional nature does not consent to hypothesizing causal relationships among variables, while the absence of a control group prevents from interpreting the results univocally as specific for eating disorders rather than linked to a general psychiatric vulnerability. Finally, it is essential to note the absence of specific measures for EDs, which may limit the interpretation of the findings. Additionally, the fact that most of the measures used were self-reported is noteworthy. In contrast, the fact that most of the measures used were self-reported. Reported specific aspects; therefore, the data may be biased. It is essential to note the absence of specific measures for EDs, which may limit the interpretation of the findings. Additionally, since most of the measures used were self-reported, patients might have underreported specific aspects, potentially biasing the data due to social desirability.

## What is already known on this subject?

- Eating disorders are associated with a higher suicide risk
- Several proximal and distal factors predictive of suicide risk are also present in patients with eating disorders (i.e., childhood trauma, depressive symptoms, hopelessness, emotion dysregulation)
- However, the interaction between these risk factors has been scarcely studied in clinical populations with eating disorders

## What does this study add?

- This study identifies variables associated with suicidal ideation in patients with EDs, such as childhood sexual abuse and loss of meaning
- This study focuses on the importance of investigating both distal factors from patient’s history and current, subjective psychological elements that can contribute to suicide risk
- The results of this study highlight the need for a thorough assessment of patients with eating disorders that goes beyond a simple investigation of eating patterns and consider a wider range of psychological factors

## Ethics approval and consent to participate

The study protocol was approved by the Local Ethics Committee, and the study adhered to the Declaration of Helsinki. Informed consent was obtained from all individual participants included in the study.

## Data Availability

No datasets were generated or analyzed during the current study.
